# Intrapelvic Localised Hydatid Cyst

**DOI:** 10.7759/cureus.2956

**Published:** 2018-07-10

**Authors:** Murat F Ferhatoglu, Ali I Filiz

**Affiliations:** 1 General Surgery, Okan University Medical Faculty, Istanbul, TUR

**Keywords:** echinococcus granulosus, pelvic, hydatic cyst

## Abstract

Hydatid cysts, caused by Echinococcus granulosus, is an important health problem in endemic areas. The disease can localize most commonly in the liver and lungs. Primary pelvic involvement is a rare condition. In this case report, we aimed to present a 75-year-old male patient with a pelvic mass, which was diagnosed preoperatively as a hydatid cyst.

## Introduction

Hydatid cyst disease is endemic to the Middle East, South America, East Africa, and the Mediterranean countries [1]. Sheep, pig, goat, and man are intermediate hosts, harboring the larval stage. Adult forms of Echinococcus granulosus parasites live in the intestines of dogs. The eggs formed by the adult forms are absorbed by people's mouths when they eat contaminant foods that are infested with dog feces. Once the eggs open in the human stomach, they spread through the blood and can form cystic structures in all organs [2]. The most frequently observed organs with this cystic formation are the liver and lungs [3]. Primary pelvic involvement is a rare condition. In this case report, we aimed to present a 75-year-old-male patient with a pelvic mass that was diagnosed preoperatively as hydatid cyst disease.

## Case presentation

**Figure 1:**
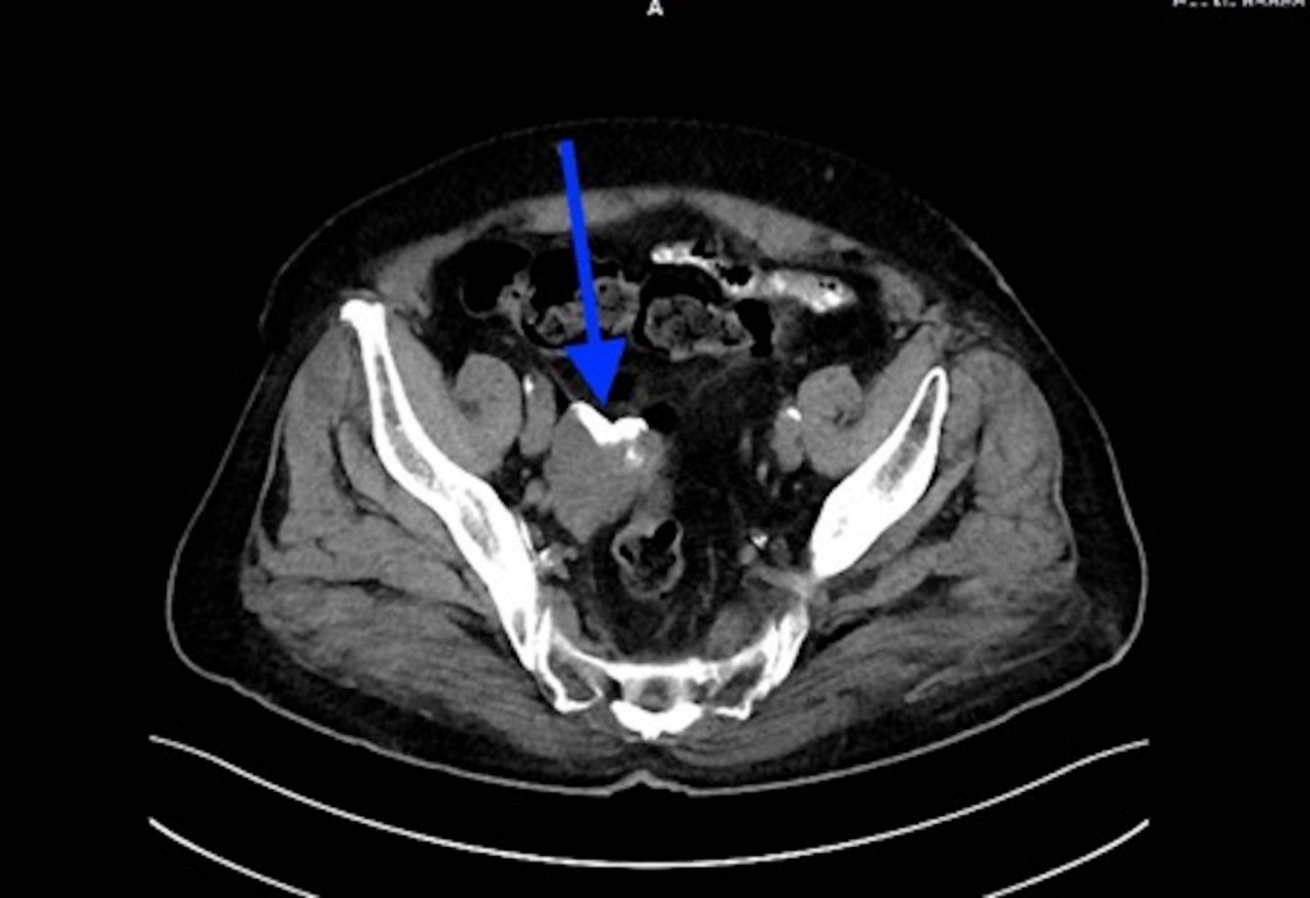
Computed tomography image, 5 x 4 cm calcified mass (blue arrow)

**Figure 2:**
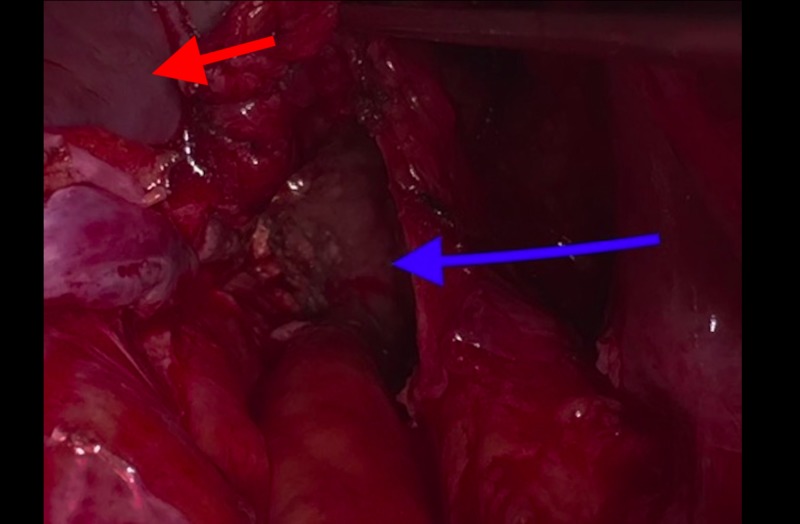
Deep pelvic localization of hydatid cyst (blue arrow), urinary bladder (red arrow)

**Figure 3:**
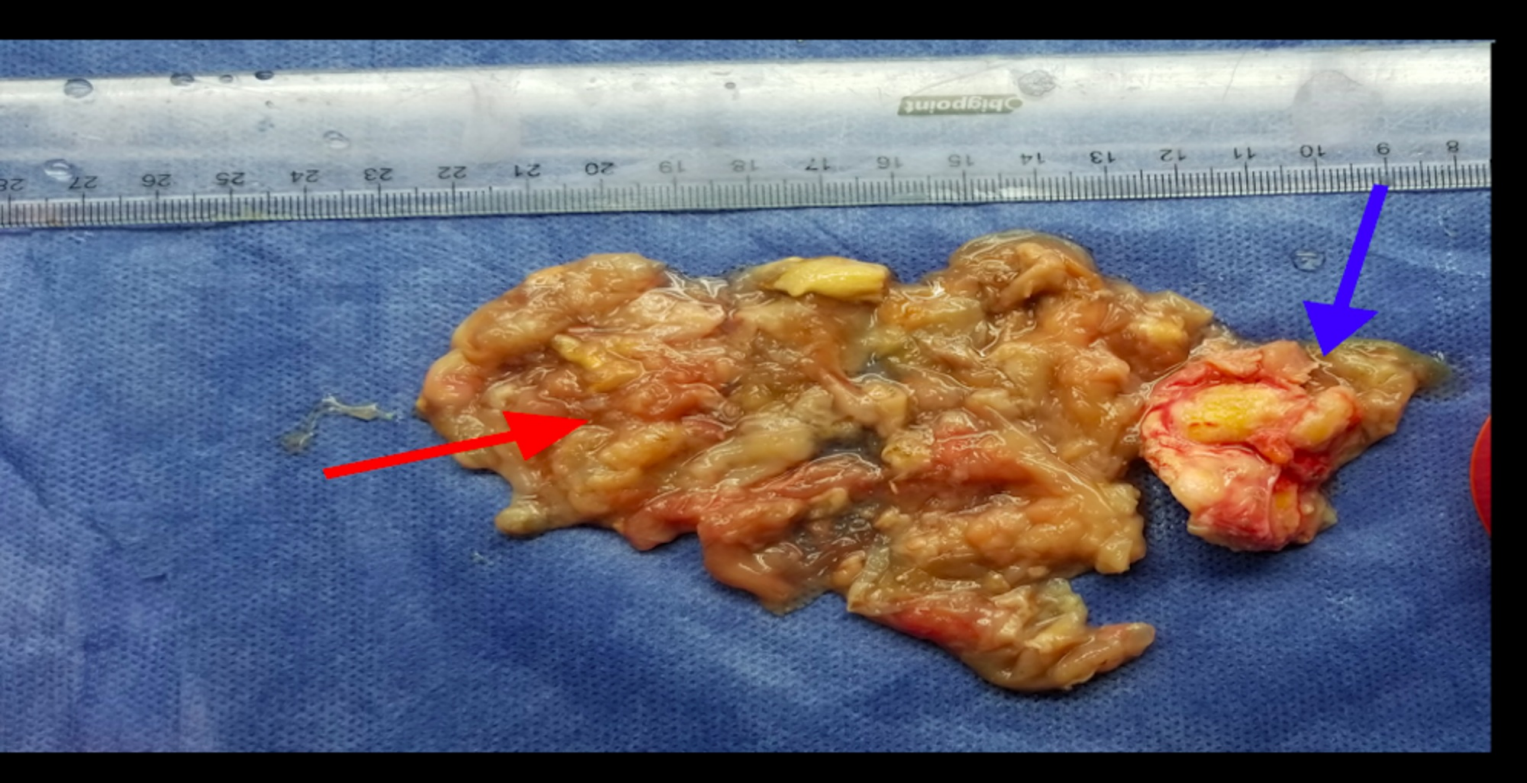
Excised specimen of cyst. Calcified cyst wall (blue arrow), dead membranes of daughter vesicles (red arrow)

A 75-year-old Iraqi male presented to the surgery clinic with chronic abdominal and right inguinal pain. He did not have a history of any surgery or trauma and was taking amlodipine 10 mg for hypertension. On examination, the patient’s blood pressure was 135/95 mm Hg, heart rate was 62 bpm, and body temperature was 36.8°C. His abdominal examination did not reveal any signs of rigidity, rebound, or pulsatile mass. The laboratory examination findings were: leukocyte: 7300/mm3(4600-10200/mm3), c-reactive protein: 2.7mg/dL (0-5 mg/dL), potassium: 3.8 mmol/dL (3.5-5.1 mmol/dL), aspartate aminotransferase: 34U/L (5-34 U/L), and alanine aminotransferase: 205 U/L (0-55 U/L). Plain abdominal X-ray and ultrasonography did not reveal any abnormality. Intravenous contrast-enhanced computed tomography (CECT) scan revealed a calcified mass that was 60x52 mm in size and localized between the right internal iliac artery and urinary bladder (Figure 1). The Tru-cut biopsy of the mass was undiagnostic and did not reveal if the mass was benign or malignant. It was decided to take the surgical approach. An explorative laparotomy was performed with a vertical midline incision. A lesion that was 5x4 cm in diameter and localized in the retroperitoneal area, between the right internal iliac artery and the urinary bladder, was excised (Figures 2-3). The frozen section pathological examination of the mass revealed that it was a pelvic hydatid cyst. Definitive pathological results also confirmed the results of the frozen section. On the first day after the surgery, a liquid diet was given. His vital signs were normal on follow-up, and he was discharged uneventfully on the fourth day of the operation. Albendazole 200 mg/day was prescribed to the patient.

## Discussion

Echinococcus granulosus is a parasite that causes hydatid cyst disease. This infection is usually observed in people who have close contact with dogs and sheep [4]. The disease is endemic in the Middle East, South America, East Africa, and the Mediterranean countries. The most common localizations of this disease are the liver and lung. Hydatid cyst disease is rarely observed in the pelvis, and the incidence is between 0.20% and 2.25% [5]. The cases are usually secondary to the rupture of a hydatid cyst localized in the liver. Primary pelvic hydatid cyst is rarely found and a small number of pelvic localized hydatid cyst cases have been reported in the literature [6]. These events may occur when the parasite reaches the pelvis by hematogenous or through the lymphatic route.

Symptoms are non-specific but the main symptom is pelvic pain. Symptoms are related to pressure. Menstrual irregularity in females and infertility can be observed [7]. On physical examination, there are no specific findings. A palpable pelvic mass can be detected [5]. Ovarian cyst, mesenteric cyst, gastrointestinal duplication cyst, cystadenoma, and lymphangioma should be kept in mind in the differential diagnosis [8]. Cysts can sometimes spontaneously rupture, and a serious anaphylactic reaction may develop in this situation.

Serological tests may be helpful in the diagnosis, but even they are not a 100% reliable [9]. The disease can be detected by imaging methods. Because of its low cost and easy accessibility, ultrasonography is preferred as the first imaging method [7]. Computed tomography may provide better information about cyst morphology and is more successful in showing the calcification and morphology of cysts [10]. Magnetic resonance imaging (MRI) may provide some advantages over computed tomography (CT) for identifying residual lesions and recurrences, especially after surgery [3].

Mebendazole or albendazole treatment is successful in the majority of the cases, but medical therapy should not be preferred as the primary treatment method except in cases where the patient is not suitable for the surgical approach or the cyst size is small or deeply located. Surgical treatment is the most effective method. A combination of preoperative drugs with surgery and postoperative medical therapy is the preferred regime [9]. It has been reported that the use of mebendazole or albendazole reduces the risk of recurrence from 80% to 10% and albendazole is the most effective medical treatment agent for Echinococcus granulosus [11].

En bloc resection without rupture or spreading of the daughter cyst is the recommended treatment strategy [12]. The use of scolicidal agents before the cyst membrane is opened during surgery is recommended to kill daughter vesicles, for preventing the spread of the cyst fluid, which may cause an anaphylactic reaction [13].

Albendazole or mebendazole should be used in postoperative treatment. Albendazole given preoperatively in a dose of 10 mg/kg/day for one month kills most of the protoscoleces within the hepatic hydatid cyst. Ultrasonography and serological tests are useful in postoperative follow-ups [14].

## Conclusions

A primary pelvic hydatid cyst is rarely seen, and it should be considered in the differential diagnosis of pelvic masses, especially in those who live in or travel to endemic areas. In addition, the disease might not be diagnosed in the preoperative period despite all radiological and laboratory methods.
